# KRAS-driven immune exclusion in pancreatic ductal adenocarcinoma: mechanisms and therapeutic reversal

**DOI:** 10.1093/oncolo/oyag160

**Published:** 2026-06-02

**Authors:** Kenneth A Kern

**Affiliations:** Skaggs School of Pharmacy and Pharmaceutical Sciences, University of California San Diego, San Diego, CA, USA

**Keywords:** KRAS, pancreatic ductal carcinoma, tumor microenvironment, immunotherapy, autophagy, tumor escape

## Abstract

Pancreatic ductal adenocarcinoma (PDAC) remains largely resistant to immunotherapy, with response rates below 5%. Emerging evidence suggests that immune exclusion in PDAC is actively maintained by oncogenic KRAS signaling. Mechanistic studies implicate three coordinated processes: autophagy-mediated degradation of MHC-I and impaired antigen presentation, expansion of suppressive myeloid populations, and desmoplastic stromal remodeling that restricts cytotoxic T-cell access to malignant epithelium. These barriers are at least partially reversible. Inhibition of autophagy restores surface MHC-I expression and re-enables antigen-dependent CD8+ T-cell killing. Suppression of KRAS pathway signaling remodels the myeloid compartment, alters stromal architecture, and increases intratumoral CD8+ T-cell infiltration in experimental systems. Recent studies further demonstrate that combining allele-specific KRAS inhibition with multi-arm immunotherapy can produce durable responses in resistant PDAC models. Spatial profiling data also suggest that immune architecture differs by KRAS allele, with KRAS G12D tumors more frequently exhibiting stromal T-cell segregation and KRAS G12R tumors demonstrating altered immune proximity and metabolic dependence. Together, these findings support a model in which KRAS-targeted therapy functions not only as tumoricidal therapy but also as immune conditioning of the tumor microenvironment. Translating this biologic reversibility into durable clinical benefit will require rational, barrier-specific, and allele-informed combination strategies.

Implications for Practice Immune checkpoint blockade in advanced PDAC yields poor survival, highlighting the need for new therapeutic strategies. Mechanistic evidence indicates that KRAS inhibition may function not only as tumoricidal therapy but also as immune conditioning by partially reversing antigen loss, myeloid suppression, and stromal exclusion. Preclinical studies show that immune remodeling occurs during active KRAS-targeted therapy, suggesting that integration with immunotherapy may be optimized by aligning treatment with biologic effects on antigen presentation and immune cell composition. Durable benefit will likely require combination strategies that address these barriers simultaneously and account for the timing of immune remodeling during KRAS pathway inhibition.

## Introduction

Pancreatic ductal adenocarcinoma (PDAC) is widely recognized as an immune-excluded (“cold”) tumor.^1–4^ The reasons for immune exclusion are multifactorial. PDAC carries a relatively low mutational burden and therefore expresses fewer neoantigens for effector T lymphocytes (T cells) to recognize. It also develops an immunosuppressive tumor microenvironment (TME), characterized by expansion of myeloid-derived suppressor cells (MDSCs), tumor-associated macrophages (TAMs), and regulatory T cells (Tregs), which actively inhibit effector T-cell function. In addition, the dense desmoplastic stroma limits T-cell infiltration. Histologic studies demonstrate that effector T cells frequently marginate along stromal borders rather than infiltrating malignant epithelial nests.^1^^,^^5^ These biologic features are reflected clinically by persistent resistance to immunotherapy.

This pattern of immune suppression and clinical resistance to immunotherapy has been assumed to reflect a fixed biological property of PDAC, largely because diverse immunotherapies, including checkpoint blockade, cellular therapies, cancer vaccines, and microenvironment-targeted strategies, have failed to meaningfully modulate the TME and shown minimal clinical activity.^3^^,^^4^^,^^6–8^ However, if immune exclusion is understood not as a passive trait but as a tumor-driven process dependent on persistent oncogenic signal transduction, and in PDAC predominantly on KRAS signaling, then inhibition of this pathway may reverse the excluded microenvironment and improve responsiveness to immunotherapy.

**Figure 1:**
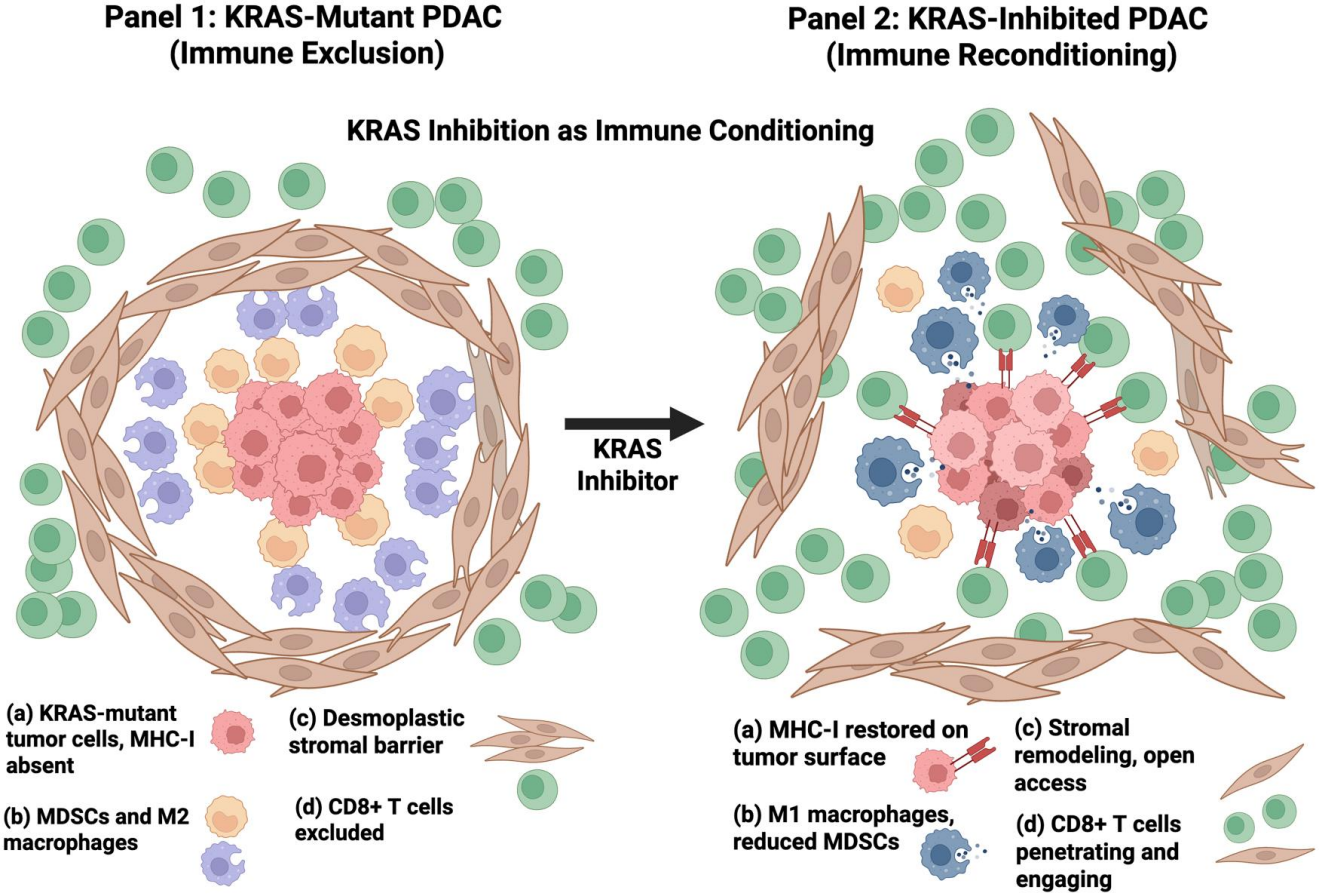
KRAS-driven immune exclusion in pancreatic ductal adenocarcinoma and therapeutic reversal. Panel 1 (left): KRAS-Mutant PDAC. Oncogenic KRAS signaling sustains immune exclusion through three convergent mechanisms: (a) autophagy-mediated degradation of MHC class I on the surface of tumor cells, impairing antigen presentation; (b) expansion of immunosuppressive myeloid-derived suppressor cells (MDSCs) and M2-polarized macrophages surrounding the tumor nest; and (c) dense desmoplastic stromal remodeling that physically blocks CD8+ T-cell access to tumor epithelium. (d) CD8+ T cells are excluded at the stromal boundary, unable to reach malignant epithelium. Panel 2 (right): KRAS-Inhibited PDAC. KRAS pathway inhibition partially reverses each barrier: (a) MHC class I is restored on the tumor cell surface, permitting T-cell receptor-dependent recognition; (b) myeloid populations are reprogrammed, with macrophages repolarizing from M2 to M1 anti-tumor phenotype and MDSCs reduced in number; (c) stromal architecture loosens, permitting passage through the fibroblast barrier; (d) CD8+ T cells penetrate the stroma and engage the tumor nest, with T-cell receptor-MHC class I contacts indicated. The arrow between panels indicates KRAS inhibition acting as immune conditioning in addition to direct tumoricidal therapy. Created in BioRender. Kern, K. (2026).

Oncogenic KRAS mutations, present in more than 90% of PDAC cases, are the strongest candidates for drivers of the oncogenic signaling that maintains immune exclusion in PDAC.^9^ The sections that follow examine evidence linking KRAS signaling to impaired antigen recognition (Section I.A), recruitment of suppressive myeloid populations (Section I.B), and stromal remodeling that restricts immune access (Section I.C), and then consider pharmacologic reversal, adaptive resistance, and the rationale for combining KRAS-targeted therapy with immunotherapy (Section II), as illustrated in Figure 1.

Although this review focuses on KRAS-driven mechanisms, immune exclusion in PDAC is multifactorial, and other biologic features contribute to immune resistance. For example, PDAC harbors a low tumor mutational burden relative to immunotherapy-responsive malignancies, limiting the neoantigen repertoire available for T-cell recognition.^10^ The gut and intratumoral microbiome has emerged as a modulator of PDAC immunobiology; long-term survivors of PDAC demonstrate greater microbial diversity within the tumor, and the composition of intratumoral bacteria has been associated with differential immune cell infiltration.^11^ Stromal heterogeneity, including regional variation in cancer-associated fibroblast subtypes and extracellular matrix density, further modulates immune access in ways that are not entirely KRAS-dependent.^12^ These factors represent additional contributors to immune resistance; however, the near-universal prevalence of KRAS mutations in PDAC and the recent availability of direct KRAS inhibitors, including sotorasib and adagrasib,^13^^,^^14^ make this pathway a rational focus for the therapeutic strategies discussed in this review.

## Methods: literature selection

This focused narrative review examines the biological and therapeutic implications of oncogenic KRAS in pancreatic ductal adenocarcinoma (PDAC), with emphasis on its role in innate and adaptive immune dysfunction. Relevant literature was identified through targeted searches of PubMed and Google Scholar using combinations of the terms KRAS, pancreatic ductal adenocarcinoma, immune exclusion, tumor microenvironment, and KRAS inhibition. Conference abstracts from recent American Association for Cancer Research (AACR) annual meetings were included when they provided mechanistic insights not yet available in peer-reviewed publications. Digital search and cross-referencing tools were used to facilitate literature identification, followed by manual review and selection of primary and review articles based on relevance to the mechanistic and clinical questions addressed. This review is selective and hypothesis-driven rather than a systematic or exhaustive survey of the literature.

## Section I. KRAS-driven immune exclusion in PDAC

### Section I.A. KRAS-driven impairment of antigen recognition and T-cell function

Impaired antigen recognition represents the first major mechanism by which oncogenic KRAS signaling limits local immune function in PDAC. In KRAS G12D-driven genetically engineered mouse models (GEMMs) of PDAC and KRAS-mutant human cell lines, Yamamoto et al. demonstrated that reduced surface MHC class I expression resulted not from mutation or deletion of antigen presentation genes, but from active autophagy-mediated degradation of MHC-I molecules.^15^ Disruption of key autophagy genes, including ATG3 or ATG7, increased plasma membrane MHC-I by approximately 1.5-fold and restored antigen-dependent CD8+ T-cell-mediated killing.^15^ In orthotopic KRAS-mutant murine PDAC models, autophagy inhibition reduced tumor weight from approximately 0.45 g to 0.1 g (*P* = 0.0002) and increased intratumoral CD8+ T cells from approximately 2% to 8.5% of CD45+ cells.^15^ These findings establish that antigen presentation in KRAS-driven PDAC is actively suppressed through protein degradation while the MHC-I genes themselves are neither mutated nor deleted. Restoration of antigen visibility alone, however, does not overcome concurrent myeloid-mediated suppression or stromal exclusion, which constitute additional barriers to effective immunity.

### Section I.B. KRAS-driven recruitment of myeloid suppressor populations

Oncogenic KRAS promotes another major mechanism of immune evasion by inducing tumor-derived GM-CSF, which drives expansion of immunosuppressive myeloid-derived suppressor cells (MDSCs) that inhibit cytotoxic T-cell function. In Kras G12D-driven pancreatic tumor models derived from genetically engineered mice, GM-CSF expression increased 14- to 18-fold at the mRNA and protein levels, with circulating GM-CSF rising approximately fivefold (*P* = 0.0031). This cytokine surge expanded Gr1+CD11b+ MDSCs from approximately 2% to nearly 18% of CD45+ immune cells within the tumor microenvironment, resulting in the absence of cytotoxic CD8+ T cells from established malignant lesions. The resulting immune microenvironment in KRAS-driven PDAC is myeloid-rich and CD8-poor, with the limited cytotoxic T-cell population further constrained by physical exclusion from direct contact with malignant epithelium.^5^

These experimental findings have correlates in human disease. In resected pancreatic cancer specimens, 14 of 16 demonstrated GM-CSF expression in PanIN (premalignant pancreatic intraepithelial neoplasia) lesions, whereas benign pancreatic tissue was negative for GM-CSF expression. In complementary murine experiments, genetic silencing of GM-CSF reduced MDSC accumulation by roughly 80%, decreased tumor implantation and lesion volume fivefold, and restored significant CD8+ T-cell infiltration. Depletion of CD8+ T cells abolished this antitumor effect, confirming that tumor regression following GM-CSF suppression depended on restored cytotoxic T-cell activity rather than direct tumor cell effects.^16^ This KRAS-driven myeloid expansion is not unique to PDAC; similar chemokine-mediated MDSC recruitment has been demonstrated in KRAS-mutant colorectal cancer models, establishing KRAS-driven myeloid recruitment as a conserved feature of oncogenic KRAS biology across gastrointestinal malignancies.^17^

### Section I.C. KRAS-driven stromal remodeling and physical immune exclusion

Oncogenic KRAS contributes to immune exclusion in PDAC by reprogramming tumor-associated fibroblasts to create both a structural barrier and a cytokine-mediated suppressive environment that limits cytotoxic T-cell access to tumor cells. In PDAC models, KRAS-mutant tumor cells induce two functionally distinct fibroblast states: one that produces dense extracellular matrix and another that secretes cytokines.^18^ The matrix-producing fibroblasts deposit collagen and other matrix proteins that prevent CD8+ T cells from physically penetrating through the stroma to reach malignant epithelial cells. The cytokine-secreting fibroblasts markedly increase IL-6 expression approximately 40-fold and upregulate CXCL1 and CXCL2, all of which recruit suppressive myeloid cells into the tumor microenvironment. In this setting, KRAS-driven tumor-fibroblast signaling blocks cytotoxic T-cell entry and reinforces myeloid-mediated immune suppression, further restricting effective tumor cell killing. The result in PDAC is the dense desmoplastic stroma characteristic of this malignancy. This desmoplastic stroma elevates interstitial fluid pressure to levels that compress intratumoral vasculature, impairing both drug delivery and immune cell access.^19^^,^^20^

In resected human PDAC specimens, immune exclusion has been demonstrated at the microscopic interface between tumor cells and surrounding stroma. Ene-Obong et al.^5^ demonstrated that CD8+ T cells were present within the stromal compartment but were scarce within 100 micrometers of tumor epithelium, the so-called juxtatumoral interface.^5^ Juxtatumoral CD8+ density below 0.05 cells per high-power field was observed in most PDAC cases; tumors at or below this threshold were associated with significantly decreased survival (*P* = 0.045), whereas overall stromal CD8+ density did not reach statistical significance for survival (*P* = 0.06).^5^ These findings demonstrated that cytotoxic T cells were not absent from PDAC but were physically restricted from reaching tumor epithelium, where effector function is critical to antitumor activity. Together, elevated interstitial pressure and stromal barriers limit CD8+ T-cell access to malignant epithelium, establishing physical exclusion as a third mechanism of immune resistance in KRAS-driven PDAC.

Spatial profiling of human PDAC specimens has demonstrated that KRAS-mutant tumors exhibit an abnormal distribution of immune cells within the tumor microenvironment, with effector T cells segregated from malignant epithelium rather than in contact with it. McIntyre et al^21^ found that this segregation pattern varies by KRAS allele, with G12D tumors exhibiting T-cell-rich niches physically separated from tumor cells and G12R tumors showing closer tumor-immune proximity but reduced T-cell density.

In Sections I.A through I.C, we have described three downstream consequences of oncogenic KRAS signaling that restrict antitumor immunity: impaired antigen presentation, expansion of suppressive myeloid cells, and physical exclusion of cytotoxic T cells. The following section evaluates whether these KRAS-driven barriers, illustrated in Figure 1, are therapeutically reversible.

## Section II. Reversal of KRAS-Activated immune suppression

If oncogenic KRAS signaling actively maintains immune exclusion in PDAC, the critical question is whether these mechanisms can be therapeutically reversed. Clinical data addressing this question in PDAC remain limited, but preclinical studies demonstrate that multiple components of KRAS-driven immune suppression are pharmacologically modifiable.

As described in Section I.A, autophagy inhibition restores MHC-I expression on KRAS-mutant PDAC tumor cells and re-enables antigen-dependent CD8+ T-cell killing, with substantially improved response rates when combined with checkpoint blockade.^15^

In addition to restoring antigen presentation, KRAS inhibition also reshapes the myeloid compartment of the tumor microenvironment. Norgard et al. blocked the KRAS pathway in syngeneic KRAS-mutant murine tumor models using a combination of SOS1 inhibition (BI-3406) and MEK inhibition (trametinib). These investigators observed a 2-to-3-fold increase in intratumoral CD8+ T cells with enhanced cytotoxic function. However, single-cell RNA sequencing revealed that SOS1i+MEKi simultaneously increased M2 macrophage polarization and impaired dendritic cell maturation, creating a paradoxically immunosuppressive myeloid environment despite improved T cell infiltration. Reversal of this M2 polarization required the addition of a CD40 agonist,^23^ and durable complete responses with immune memory were achieved only when SOS1i+MEKi was combined with CD40 agonism and checkpoint blockade.^22^ Additional work has demonstrated that RAS(ON) multi-selective inhibition remodels cancer-associated fibroblast subtypes and extracellular matrix composition in pancreatic cancer, indicating that KRAS pathway suppression also alters the stromal architecture of the tumor microenvironment.^24^

Consistent with the need for immunologic co-targeting demonstrated by Norgard et al., Liu et al. found that the KRAS G12D inhibitor MRTX1133 produced tumor regression in a KRAS G12D-driven PDAC mouse model (iKPC) but did not achieve durable complete responses when used alone. When MRTX1133 was combined with a multi-agent immunotherapy regimen consisting of a CXCR1/2 inhibitor (to block myeloid recruitment), anti-LAG3 (to reduce T-cell exhaustion), and anti-4-1BB (to enhance T-cell co-stimulation), durable complete responses were observed in 36% of treated mice bearing established tumors. In contrast, combination with anti-PD-1 alone was insufficient to achieve similar durability.^25^ These findings, along with those of Norgard et al., indicate that effective reversal of KRAS-driven immune suppression may require simultaneous correction of antigen presentation, myeloid recruitment, and T-cell exhaustion rather than single immune pathway modulation.

McAndrews et al. evaluated BI-2493, a pan-KRAS inhibitor with activity against multiple mutant alleles including G12C, G12D, and G12V, across a range of experimental PDAC systems including cell lines, patient-derived xenografts, syngeneic orthotopic models, and genetically engineered mouse models. Pan-KRAS inhibition suppressed tumor growth and prolonged survival across these systems. In immune-replete models, treatment increased intratumoral CD8+ effector T cells and reduced myeloid cell infiltration, with responsiveness to immune checkpoint blockade. Long-term treatment selected for resistance including YAP signaling upregulation, reinforcing the need for combination strategies and highlighting the importance of allele-agnostic investigation in PDAC, where G12D, G12V, and G12R predominate.^26^

The impact of KRAS inhibition on tumor immunity varies by KRAS allele, co-mutations, and baseline T-cell infiltration. For example, tumors with concurrent TP53 mutations tend to exhibit greater immune infiltration and checkpoint responsiveness, whereas co-mutations in STK11 or KEAP1 confer immune resistance that persists despite KRAS pathway inhibition.^27^ In PDAC, where approximately 40% of tumors harbor KRAS G12D mutations,^21^ PD-L1 expression is rarely above 10% ^28^ and dense stroma limits immune cell access, multi-barrier intervention is highly likely to be required. Broderick et al. demonstrated that combining a RAS(ON) multi-selective inhibitor with the CDK4/6 inhibitor palbociclib drove drug-tolerant, “persister” cells into a senescent-like state in PDAC models. When CD40 agonism was added as a third agent, CD4+ T cells engaged in immune surveillance that maintained residual tumor cells in a non-proliferative state, producing durable tumor control dependent on IFN-gamma signaling rather than direct T-cell cytotoxicity.^29^

Additional preclinical studies reinforce the central role of immune engagement in KRAS-targeted therapy. Orlen et al. demonstrated that tumor regressions achieved with RAS(ON) multi-selective inhibition in preclinical PDAC models are T-cell dependent, establishing immune remodeling as a functionally required component of the antitumor response.^30^ Barbacid and colleagues showed that KRAS inhibition combined with EGFR and STAT3 blockade achieved effective regression and prevented resistance emergence.^31^

An important caveat applies to these preclinical findings. The murine models that demonstrate immune remodeling following KRAS pathway suppression differ from human PDAC in several respects that may limit direct translation. Human PDAC stroma is denser and more collagen-rich than that of mouse models, with higher interstitial fluid pressures,^32^ and the proportions and functional states of myeloid populations differ between species. Additionally, the transplantable and spontaneous tumor models used in these studies employ a limited set of KRAS alleles and may not capture the full spectrum of immune phenotypes observed across KRAS-mutant human PDAC. Whether the degree of immune remodeling observed in experimental systems can be reproduced in the context of the human PDAC stroma remains an open question that will require correlative tissue analysis from ongoing clinical trials.

## Section III. Clinical strategies to overcome KRAS-driven immune exclusion

The preceding sections demonstrate that oncogenic KRAS signaling sustains immune exclusion in PDAC through three biologically distinct but interrelated mechanisms: impaired antigen presentation, expansion of suppressive myeloid populations, and desmoplastic stromal exclusion of cytotoxic T cells. Experimental systems have shown that each of these mechanisms is at least partially reversible, raising the question of whether reversal of one or more of these components can produce durable therapeutic benefit in patients.

Several clinical trials have attempted to overcome immune exclusion in PDAC by targeting the tumor microenvironment directly. The CD40 agonist sotigalimab (a CD40 agonistic antibody) was evaluated in the PRINCE trial, a randomized phase 2 study of 105 chemotherapy-naive patients with first-line metastatic PDAC who received gemcitabine/nab-paclitaxel combined with nivolumab (anti-PD-1), sotigalimab, or both. The primary endpoint of one-year overall survival was met for the nivolumab/chemotherapy arm (57.7%, *P* = 0.006 vs. historical 35%) but was not met for sotigalimab/chemotherapy (48.1%, *P* = 0.062) or sotigalimab/nivolumab/chemotherapy (41.3%, *P* = 0.223). Biomarker analyses identified treatment-specific immune correlates: survival after nivolumab/chemotherapy correlated with activated T cells at baseline, while sotigalimab/chemotherapy correlated with intratumoral CD4 T cell infiltration. No patient subset benefitting from the triple combination was identified.^33^

Another CD40 antibody investigated was mitazalimab (a human CD40 agonistic IgG1 antibody), evaluated in 57 patients in the OPTIMIZE-1 trial, a dose-finding and safety study of chemotherapy-naive patients with metastatic PDAC. Mitazalimab combined with mFOLFIRINOX produced a 40% objective response rate; the investigators concluded that these results warrant evaluation in a phase 3 randomized controlled trial.^34^ The stroma-depleting enzyme pegvorhyaluronidase alfa (PEGPH20) was evaluated in HALO 109-301, a phase 3 randomized, double-blind, placebo-controlled trial of 492 patients with hyaluronan-high metastatic PDAC. PEGPH20 added to nab-paclitaxel/gemcitabine increased the objective response rate (47% vs. 36%) but failed to improve overall survival (median 11.2 vs. 11.5 months; HR 1.00; *P* = 0.97) or progression-free survival.^35^ Despite these individual attempts to reverse immune cell suppression or desmoplasia, a common limitation of these trials is that none addressed the KRAS-driven immunosuppression that is the primary driver of immune resistance in PDAC.

As reviewed in Section II, preclinical studies demonstrate that KRAS pathway inhibition simultaneously reverses multiple components of this immunosuppressive program, including restoration of MHC-I expression,^36^^,^^37^ reduction of MDSC recruitment,^38^ and activation of senescence-associated immune programs.^39^ In murine lung cancer models, the KRAS G12C inhibitor adagrasib produced simultaneous MHC-I upregulation, MDSC reduction, and macrophage repolarization from M2 to M1 phenotype, with durable complete responses occurring only when combined with checkpoint blockade.^40^ However, KRAS G12C accounts for only approximately 1% of PDAC, and the dominant alleles are G12D, G12V, and G12R. Replicating these effects in PDAC will require allele-selective inhibitors such as MRTX1133, pan-RAS inhibitors such as daraxonrasib, or rationally designed combination regimens, and whether any will produce durable clinical benefit remains to be established in prospective trials.

## Proposed strategies for reversing KRAS-driven local immune dysfunction in clinical investigation

The clinical and preclinical evidence reviewed above identifies three distinct mechanisms by which KRAS-mutant tumor cells suppress local immune function in PDAC: loss of antigen presentation, expansion of immunosuppressive myeloid populations, and physical exclusion of T cells by desmoplastic stroma. Four clinical strategies for reversing these barriers to antitumor immunity emerge from this evidence:

### Restore antigen presentation

Restoring MHC-I expression on the tumor cell surface through autophagy inhibition is one proposed strategy for enhancing immune recognition, particularly in KRAS G12R tumors, which depend more heavily on autophagy for survival.^41^ Clinical trials testing autophagy inhibitors in PDAC would need to incorporate paired biopsies to confirm MHC-I restoration or include antigen-specific CD8+ T-cell recognition assays as pharmacodynamic endpoints.

### Reprogram myeloid suppression

As described in Section II, KRAS pathway inhibition increases T cell infiltration but does not completely resolve myeloid suppression. Norgard et al. demonstrated that SOS1/MEK inhibition paradoxically increased M2 macrophage polarization and impaired dendritic cell maturation, and durable responses required the addition of CD40 agonism.^22^ Liu et al. similarly found that durable complete responses with KRAS G12D inhibition required multi-arm immunotherapy combining myeloid-directed agents with co-stimulatory agonists.^25^ These findings indicate that clinical trials of KRAS inhibition in PDAC should incorporate myeloid-directed co-targeting, such as CXCR1/2 inhibition or CD40 agonism, with myeloid polarization profiles serving as pharmacodynamic endpoints.

A practical consideration is the cumulative drug-related toxicity seen in PDAC patients frequently debilitated by anorexia, cachexia, biliary obstruction, and prior intense chemotherapy. Combining KRAS pathway inhibitors with myeloid-directed agents and checkpoint or co-stimulatory antibodies could produce overlapping gastrointestinal, hepatic, and hematologic toxicities.^31^ To protect patient safety, early-phase trial designs should incorporate well-defined dose-modification algorithms for gastrointestinal and myelosuppressive adverse events. Sequential or staggered administration, rather than simultaneous combination, may preserve antitumor effects while reducing the probability of intolerable toxicity.

### Account for allele-specific differences in immune cell infiltration

The preceding strategies assume a uniform pattern of immune exclusion across KRAS-mutant PDAC. However, spatial profiling data from McIntyre et al., based on 20 resected PDAC specimens, suggest that the nature of immune dysfunction differs by KRAS allele.^21^ In KRAS G12D tumors (approximately 40% of PDAC), T cells were present in substantial numbers but were physically segregated from tumor epithelium by desmoplastic stroma, consistent with the classical model of immune exclusion described above.^19^ In KRAS G12R tumors (approximately 20% of PDAC), T cells were found in closer proximity to tumor cells but at significantly reduced density.

These findings, if confirmed in larger cohorts, suggest that optimal therapeutic strategies may differ by allele. G12D-predominant tumors may require interventions that reduce stromal barriers to allow existing T cells to reach the tumor, whereas G12R-predominant tumors may require strategies that expand the cytotoxic T-cell population within the tumor microenvironment. Clinical trial designs should consider KRAS allele as a stratification factor when evaluating novel regimens to reverse local immune dysfunction in PDAC.

### Identify and treat during the Immune-Permissive window

The kinetics of KRAS pathway suppression may influence the timing of immune remodeling in PDAC. In SOS1/MEK inhibition models, CD8+ T-cell infiltration peaked during maximal ERK suppression at approximately 7–10 days and then declined as signaling rebounded at 14–21 days.^22^ In certain models, RAS(ON) inhibitors demonstrated more rapid phospho-ERK recovery within 8–48 hours.^29^ Although these findings do not establish a defined immune-permissive window in humans, they suggest that the degree of immune modulation is linked to the duration of effective KRAS pathway suppression.

Translating this observation into clinical practice will require correlative studies in patients receiving KRAS inhibitors to characterize immune remodeling in human PDAC tissue. Serial biopsies should assess immune cell composition, MHC-I expression, and markers of pathway suppression (phospho-ERK, phospho-MEK, DUSP6) to determine whether reduction in pathway activity correlates with local immune remodeling and whether this relationship differs by KRAS allele. These studies will also need to define the interval between KRAS pathway suppression and onset of immune remodeling. Until these relationships are established, the optimal sequencing of KRAS inhibitors and immunotherapy in PDAC remains a subject of future investigation.

### Proposed clinical development framework

Clinical development of KRAS inhibitors in PDAC should be structured around measurable reversal of KRAS-driven immune dysfunction, using tissue-based pharmacodynamic endpoints. Clinical feasibility will depend on acceptable toxicity profiles and sequential dosing strategies suitable for patients with advanced PDAC. Future trials should also explore whether circulating biomarkers such as serum cytokine profiles, circulating tumor DNA dynamics, or peripheral blood immune cell phenotyping can serve as non-invasive surrogates for local immune remodeling.

Table 1 summarizes a proposed framework linking each of the three forms of KRAS-driven immune dysfunction to a corresponding combination strategy and measurable pharmacodynamic endpoint suitable for early-phase clinical investigation.

**Table 1:** Clinical development strategies for reversing KRAS-driven immune suppression in PDAC by using barrier-specific therapy combined with KRAS inhibition.

***Barrier to Anti-Tumor Immune Response***	Biologic Mechanism	Therapeutic Strategy in Combination with KRAS Inhibition	Potential Pharmacodynamic Endpoint (Clinical Trial Setting)
***Impaired Antigen Presentation***	Autophagy-dependent degradation of MHC-I	Autophagy inhibition (e.g., hydroxychloroquine)	Surface MHC-I (tumor biopsy)
***Myeloid Suppression***	GM-CSF/CXCR2-driven expansion of MDSCs and M2-like macrophages	CXCR1/2 inhibition ± CD40 agonism	Myeloid polarization profile (MDSC/TAM phenotype)
***Stromal Exclusion***	Desmoplasia and elevated interstitial fluid pressure (IFP) limiting CD8^+^ access	Stromal-modifying agents (e.g., hyaluronidase)	IFP or perfusion imaging

Legend: Each row represents one of the three biologically distinct barriers to antitumor immunity maintained by oncogenic KRAS signaling in PDAC. For each barrier, the table lists a therapeutic strategy designed to be combined with KRAS inhibition and a pharmacodynamic endpoint for use in early-phase clinical trials. Abbreviations: MHC-I, major histocompatibility complex class I; GM-CSF, granulocyte-macrophage colony-stimulating factor; CXCR, CXC chemokine receptor; MDSCs, myeloid-derived suppressor cells; TAM, tumor-associated macrophage; IFP, interstitial fluid pressure..

## Conclusion

KRAS signaling in pancreatic ductal adenocarcinoma sustains immune exclusion through three convergent but biologically distinct mechanisms: suppression of antigen presentation via autophagy-mediated MHC-I degradation, expansion of suppressive myeloid populations through cytokine signaling, and reinforcement of desmoplastic stroma that physically restricts cytotoxic T-cell access to tumor epithelium. The link between oncogenic KRAS signaling and each of these three mechanisms has been established in Sections I and II of this review through studies demonstrating that KRAS pathway activation directly drives MHC-I degradation,^15^ MDSC recruitment via GM-CSF secretion,^16^ and fibroblast-mediated stromal remodeling.^18^ Together, these processes generate a tumor microenvironment in PDAC that is intrinsically resistant to checkpoint blockade and other immunotherapeutic strategies.

Preclinical evidence demonstrates that each component of this KRAS-driven immune suppression may be pharmacologically modifiable. Autophagy inhibition restores surface MHC-I and re-enables antigen-dependent cytotoxicity. KRAS pathway suppression reshapes the myeloid compartment and increases CD8+ T-cell infiltration. Targeting desmoplasia reduces interstitial pressure and improves immune cell access. These findings establish that immune exclusion in PDAC is a KRAS signaling-dependent state that is potentially reversible.

Several limitations of the proposed strategies should be acknowledged. In PDAC, immune remodeling following KRAS pathway suppression may be incomplete or temporally limited, and single-agent KRAS inhibition has not consistently translated into sustained tumor control in preclinical PDAC models. Meaningful clinical activity in PDAC will likely require coordinated strategies that simultaneously address antigen visibility, myeloid suppression, and stromal exclusion, while maintaining sufficient pathway suppression to support immune reconditioning. Furthermore, different KRAS alleles produce different patterns of immune dysfunction,^21^ and allele-selective inhibitors have not yet reported clinical combination data in PDAC. Optimal therapeutic strategies may need to differ by mutation context and by which form of immune dysfunction predominates in a given tumor.

KRAS-targeted therapy in PDAC can be understood as both tumoricidal and as a method of immune reconditioning through reversal of KRAS-driven antigen suppression, myeloid expansion, and stromal exclusion. Whether sustained KRAS pathway inhibition, rational combination strategies, and biomarker-guided timing of therapy can produce meaningful clinical activity through immune reconditioning in PDAC remains a subject of future investigation.

## Data Availability

No new data were generated or analyzed in support of this research.
